# The Impact of Emotions and Hedonic Balance on Teachers’ Self-Efficacy: Testing the Bouncing Back Effect of Positive Emotions

**DOI:** 10.3389/fpsyg.2019.01670

**Published:** 2019-07-17

**Authors:** Ilaria Buonomo, Caterina Fiorilli, Paula Benevene

**Affiliations:** Department of Human Science, Libera Università Maria SS. Assunta, Rome, Italy

**Keywords:** teachers, self-efficacy, trait emotions, undoing effect, professional role

## Abstract

Emotions toward students (e.g., Chan, 2004) and professional role (e.g., O’Connor, 2008) impact teachers’ self-efficacy (TSE) beliefs. The effect of positive emotions (PEs) can be explained by the broaden and build theory, stating that the higher the PEs individuals attribute to themselves, the higher the chance to build positive aspects of the self (Fredrickson, 2001). At the same time, negative emotions (NEs) at school inversely influence TSE, reducing teachers’ confidence (Chan, 2004). Furthermore, Fredrickson et al. (2000)’s studies inform about the bouncing back effect of PEs on the detrimental effects of NEs on self-efficacy. Starting from these considerations, this study (1) evaluated the bouncing back effect of PEs on negative ones, when predicting self-efficacy; (2) verified whether emotions toward professional role moderated the bouncing back effect. Self-efficacy and emotions in teaching (MESI, Moè et al., 2010) were measured. Two hundred and seventy-two Italian secondary school teachers (*F* = 73%) were involved. PEs toward students might act as buffering factors against the detrimental effect of NEs on self-efficacy [*F*(2,270) = 26.17, *P* < 0.001, *R*^2^ = 0.199]. Finally, emotions toward students and emotions toward professional role do not interact when predicting self-efficacy. The relationships with students seem to have an highly protective effect on teachers’ mental health. At the same time, the perception of one’s own role as detached from the teaching community may have a role in justifying the non-significant effect of emotions toward professional role in the model and shed new light on intervention objectives.

## Introduction

Tschannen-Moran et al. (1998) defined teachers’ self-efficacy (TSE) as the teacher’s belief in his or her capability to organize and execute the actions required to successfully accomplish professional tasks in a specific context (Tschannen-Moran et al., 1998). Teacher beliefs about professional efficacy influence several aspects in the teaching–learning process, including classroom environment, student performance, and teacher practices (Goddard et al., 2007; Santisi et al., 2014; Zee and Koomen, 2016; Benevene et al., 2018; Granziera and Perera, 2019). Accordingly, teachers with low self-efficacy are at high risk for absenteeism, physical illness, and burnout (Zee and Koomen, 2016). Given the impact of TSE on school life, influencing factors should be individuated, to shed a new light on well-being promotion at school. Consistently with Bandura’s (1986) social-cognitive theory, Tschannen-Moran et al. (1998) individuated four main sources for TSE: vicarious experiences, sense of mastery, verbal persuasion, and physiological and emotional arousal. At the same time, the authors considered physiological states and associated emotions as less effective in predicting TSE when compared to other sources. This is due to the fact that physiological states and emotions must be carefully attended to have an effect on TSE (Tschannen-Moran et al., 1998). Several studies have shown that, when workers attend their positive emotional experiences, their self-efficacy raise (Wright and Staw, 1999; Lyubomirsky et al., 2005; Tsai et al., 2007; Staw et al., 2008; O’Malley and Gregory, 2011; Xanthopoulou et al., 2012). Furthermore, current research suggests that teachers are aware of their emotional involvement in their job. More specifically, several authors show that teachers recognize the effects of emotions and relations experienced at school on their teaching goals, experiences, and results (Sutton and Wheatley, 2003; Sutton and Harper, 2009; Borrelli et al., 2014; Fiorilli et al., 2017). Consistently, some studies report that teachers who generally experiment more positive than negative emotions (NEs) feel more confident too (Sutton and Harper, 2009).

The effect of positive emotions (PEs) on self-efficacy can be read in light of the broaden and build theory, stating that the higher the PEs individuals attribute to themselves, the higher the chance to build positive aspects of the self (Fredrickson, 2001; Fredrickson and Losada, 2005). At the same time, teaching is loaded with NEs, too, for example because of student misbehavior or low social recognition (Becker et al., 2015; Mevarech and Maskit, 2015; Frenzel et al., 2016; Fiorilli et al., 2017). The emotional correlates of such problems and conflicts at school inversely influence TSE, reducing teachers’ confidence (Chan, 2004). Therefore, it seems useful to take into account the co-occurrence between positive and NEs (Diener and Emmons, 1984), and how this may influence teacher self-efficacy.

Positive and NEs, indeed, activate different biological and behavioral patterns and are independently regulated over time (Diener et al., 1985; Watson, 2000).

How positive and NEs influence one another, when influencing self-efficacy? A possible explanation is provided by the construct of undoing effect (Fredrickson et al., 2000), interpreted as the ability of PEs to restore psychological resources in face of NEs and experiences (Gloria et al., 2013). When individuals make the most of their PEs, they are better at reducing the detrimental effects of negative affect on personal interpretations of events (Tugade and Fredrickson, 2004, 2007). Consistently, previous studies reported that positive affect mediated the relationship between NEs at work and psychological resources in teachers (Gloria et al., 2013). Recognizing PEs at work, indeed, could act as a buffer, through which the potential detrimental effect of NEs on self-efficacy would be lowered. Despite this, to the best of our knowledge, no studies have explored such effects on self-efficacy.

When studying teacher emotions, a distinction should be made between emotions toward students and emotions toward the teaching professional role (Moè et al., 2010). While many studies addressed the firsts, few studies addresses the seconds, despite showing meaningful implications for the profession (Sutton and Wheatley, 2003). More specifically, teachers’ emotions toward their role guide their practices and shape their professional identities, independently from the actual teaching experience in the classroom (O’Connor, 2008; Gonzalez-Calvo and Arias-Carballal, 2017). In other words, emotions seem a crucial point of teachers’ professional life. According to Palmer (1998), this is due to the fact that the teaching profession is at the crossroad of personal and public life. With this regard, teaching professional role involves two interacting dimensions: intrapersonal factors, such as emotions and personal history (e.g., Zembylas, 2003; Van Veen and Sleegers, 2006) and social factors, such as personal and professional relations and social representations about the profession (Holstein and Gubrium, 2000; Roa-Tampe, 2017). Teachers, indeed, spontaneously refer to emotions when asked to enumerate the main dimensions of their profession (Buonomo and Fatigante, 2017; Gonzalez-Calvo and Arias-Carballal, 2017; González-Calvo and Fernández-Balboa, 2018). This broader “emotional context,” connected with teachers’ identities, influences emotions experienced with students during classroom interactions. O’Connor (2008), indeed, argued that teachers justify their caring attitude toward students as a main part of their professional identity. At the same time, current research suggests that teachers experience several NEs toward their professional role, above all related to low social recognition of their profession and feeling of social underestimation (OECD, 2014; Buonomo et al., 2017). Teachers able to balance the contrasting emotions felt toward professional role may be better at mitigating the detrimental effects of NEs on TSE, thus strengthening the protective impact of positive classroom experiences on their sense of confidence. The ability to balance positive and NEs (namely, hedonic balance; Diener et al., 1999) regarding the role, indeed, was related to efficacy beliefs in Italian teachers (Caprara and Steca, 2006).

Overall, emotions related to students and to the teaching role can be considered as distinct sources of information about teachers’ emotional experiences. Generally speaking, the emotional acknowledgment at work predicts self-efficacy beliefs (e.g., Xanthopoulou et al., 2012). For this reason, we hypothesized that emotions toward students would explain teachers’ self-efficacy. At the same time, as teachers’ identities are embedded with emotions (O’Connor, 2008), we considered emotions toward professional role as generating a broader emotional context that could influence the predictive effects of emotions toward students. More specifically, this study aimed to verify two hypotheses:

Hypothesis 1 – positive emotions toward students mediate the detrimental effect of negative emotions toward students on self-efficacy;
Hypothesis 2 – hedonic balance related to professional role moderates the mediating effect of positive emotions, so that the higher the hedonic balance, the higher the mediating effect of positive emotions.

## Materials And Methods

Participants were 272 Italian school teachers (Females = 73%), aged 26–66 (*M* = 51.50; *SD* = 7.96) years and with 1–41 years of experience (*M* = 21.74; *SD* = 10.37). The most of them were class (91%) and permanent (89%) teachers. Data collection occurred during school meetings, with principals’ consent. Teachers were instructed by written informed consent that they could leave the study and ask for further information at any time. Teachers’ self-efficacy and emotions were assessed with the Metacognitive Questionnaires for Teachers, validated on the Italian teaching population (MESI; Moè et al., 2010). Self-efficacy was measured with 24-item, on a 10-point Likert scale (1 = not effective, 10 = totally effective) (sample item: “I am good at managing students’ oppositional behaviors”). Teachers’ PE and NE, toward students and professional role, were measured with 30 items (“how frequently do you feel these emotions when you are with your students/when you think about your profession?”), measured on a 5-point Likert scale (1 = almost never, 5 = almost always). The subscale measured 17 NEs (shame, anger, uneasiness, inadequacy, wrath, exasperation, indignation, sadness, sense of failure, guilt, resignation, irritation, antipathy, frustration, discouragement, nervousness, disappointment) and 13 PEs (cheerfulness, enthusiasm, caring, commotion, admiration, complacency, pleasure, happiness, satisfaction, fulfilment, sense of accomplishment, joy, enrichment). NE and PE are computed as total mean scores of the answers to the 17 and 13 items, respectively (Moè et al., 2010). Starting from the scales of PE and NE toward the professional role, hedonic balance related to professional role was computed as the difference between PE and NE (Diener et al., 1999). Consequently, when the hedonic balance is high, the respondent perceives more PE than NE. Reliability was examined by estimating Cronbach’s alpha and McDonald’s omega (McDonald, 1999), as the last has been advocated as more informative than Cronbach’s alpha (Revelle and Zinbarg, 2009), especially for heterogeneous scales. Omega is an indicator of general factor saturation, interpreted as the precision with which scale scores estimate a latent variable common to all test items. Overall, Cronbach’s alphas ranged 0.835 (NE toward professional role) to 0.975 (PE toward professional role), while McDonald’s Omegas ranged from 0.92 (NE toward professional role and NEs toward students) to 0.95 (PE toward professional role).

The associations among self-efficacy, sociodemographic variables, student-related emotions, and role-related hedonic balance were calculated with Pearson’s product-moment correlations. As sociodemographic variables did not correlate with other variables, these were not included in the analyses. Two multiple regression analyses were conducted: (1) Hypothesis 1 (PEs toward students mediate the detrimental effect of NEs toward students on self-efficacy) was verified with a mediation model, in which beta weights are used to measure direct and indirect effects and bootstrap method (5000 samples) is used to test the effect size of the indirect effect; (2) Hypothesis 2 (hedonic balance related to professional role moderates the mediating effect of PEs) was verified with the beta weight of the interaction between hedonic balance and PE toward students. Variables were mean centered prior to the formation of the interaction term (Aiken and West, 1991), to differentiate the moderating effect at low, medium, and high levels of hedonic balance. Predicted probability plots, residuals scatterplots, and variance inflation factor (VIF) < 5, and Tolerance > 0.80 criteria were used to test, respectively, the normality, homoscedasticity, and non-multicollinearity assumption for regression. All the variables for all the regression models fulfilled the assumptions. Finally, a *p* > 0.001 Mahalanobis’ distance criterion was used to identify and skip multivariate outliers. Analyses were run with the PROCESS macro (Hayes, 2012) for IBM SPSS (vr. 23) and with the *psych* package in R (vr. 3.5.3) (Revelle, 2016).

## Results

Table 1 shows mean and standard deviation values and correlations among self-efficacy, student-related PE and NE, and role-related hedonic balance. All the associations were significant (*p* < 0.01). Overall, the higher the PEs perceived with students, the higher the hedonic balance related to professional role, the higher the perceived self-efficacy.

**TABLE 1:** Mean, standard deviations, and correlations among variables.

	***M***	***SD***	**Self-efficacy**	**Student-related negative emotions(NEs)**	**Student-related positive emotions (PEs)**	**HB-professional role**
Self-efficacy	7.28	0.89	–			
Student-related NEs	1.70	0.51	–0.371^∗∗^	–		
Student-related PEs	3.60	0.64	0.361^∗∗^	–0.347^∗∗^	–	
HB-professional role	1.58	1.13	0.337^∗∗^	–0.581^∗∗^	0.652^∗∗^	–

*^∗∗^*p* < 0.01. HB, hedonic balance.*

Table 2 shows the simple mediation model. As expected (Hypothesis 1), student-related PEs mediated the relationship between student-related NEs and self-efficacy [*F*_(2,270)_ = 26.17, *p* < 0.001, *R*^2^ = 0.199]. The effect of student-related NEs on self-efficacy was significant [*b* = −0.4918, *t*(273) = −5.0161, *p* < 0.001], as well as the effect of student-related PEs on self-efficacy [*b* = 0.3681, *t*(271) = 3.73, *p* < 0.001]. The indirect effect was significant (95% CI = −0.292 to −0.093; bootstrapped unstandardized indirect effect = −0.161, bootstrapping run on 5000 samples). Overall, results show a partial mediation of PEs in the relationship between NEs and self-efficacy.

**TABLE 2:** Regression results for simple mediation.

**Variable**	**B**	***SE***	**t**	**p**
**Direct and total effects**				
Self-efficacy regressed on NEs toward students	–0.65	0.10	–6.83	0.000
PEs toward students regressed on NEs toward students	–0.44	0.07	–6.6	0.000
Self-efficacy regressed on PEs toward students controlling for NEs toward students	0.37	0.10	0.37	0.000
Self-efficacy regressed on NEs toward students controlling for PEs toward students	–0.49	0.10	–5.02	0.000

	**Value**	***SE***	**z**	**p**

**Bootstrap results for indirect effect**				
Effect	–0.16	0.48	–0.27	–0.08

*Unstandardized regression coefficients are reported. Bootstrap sample size: 5000. LL, lower limit; CI, confidence interval; UL, upper limit.*

Table 3 shows the moderated mediation model (or indirect conditional effect; Preacher et al., 2007). Contrarily to Hypothesis 2, the role-related hedonic balance did not moderate the mediation of student-related PEs on the relationship between student-related NEs and self-efficacy [interaction effect was *b* = −0.08, *t*(271) = −1.35, NS].

**TABLE 3:** Regression results for conditional indirect effect.

**Predictor**	***B***	***SE***	***t***	***p***
**PEs toward students**				
Constant	0.75	0.13	5.85	0.000
NEs toward students	–0.44	0.07	–6.11	0.000
**Self-efficacy**				
Constant	8.14	0.21	39.33	0.000
PEs toward students (PEs)	0.35	0.10	3.52	0.000
NEs toward students (NEs)	–0.49	0.12	–4.15	0.000
Hedonic balance related to professional role (HBpr)	0.00	0.07	0.05	0.958
PEs × HBpr	–0.08	0.06	–1.35	0.179

**N* = 272. Unstandardized regression coefficients are reported. Bootstrap sample size: 5000.*

## Discussion

Our findings showed that PEs toward students partially mediate the relationship between NEs toward students and self-efficacy in a sample of Italian teachers, confirming our first hypothesis. At the same time, our results showed that the hedonic balance toward professional role did not influence the effect of PEs on self-efficacy, contrarily to our second hypothesis.

Overall, this study confirmed the role of emotions in predicting teaching self-efficacy, strengthening social-cognitive theory statements about the role of emotional arousal in predicting efficacy beliefs (Bandura, 1986; Tschannen-Moran et al., 1998).

According to our first hypothesis, when teachers frequently experience NEs toward their students, they are at higher risk for believing to be not effective in the classroom. Emotional labor is a possible explanation for this finding, as it may have influenced teachers’ answers. Emotional labor is the strain emerging from the contrast between felt emotions and organizational rules about emotion expression (Schutz and Lee, 2014). In other words, teachers may feel they are not allowed to express certain emotions because of the school’s rules about emotional disclosure (Yin and Lee, 2012). According to the emotional labor framework, indeed, the higher the emotions felt but not expressed, the higher the stress and burnout risk (Yin and Lee, 2012). At the same time, and contrarily to our results, other studies (Sutton and Harper, 2009) showed that teachers tend to express or fake anger toward their students in order to maintain discipline and that, by means of this strategy, they feel more effective at managing their classroom. To better understand these apparently contrasting results, the type of studied emotions should be considered. Studies about teachers’ emotions, indeed, usually address basic NEs (Sutton and Harper, 2009; Yin and Lee, 2012). In our study, teachers answered about their complex, self-conscious emotions (e.g., sense of failure, guilt, resignation, frustration, discouragement; Tracy and Robins, 2007), linked to experiences of loss or blame (Thamm, 2006). This may explain the reduction of self-efficacy. Individuals with high negative self-conscious emotions, like blame, guilt, and discouragement, are at higher risk to experience low self-esteem (Kuppens and Van Mechelen, 2007). Therefore, it is likely that these emotions may have a role in reducing self-efficacy, too.

According to our first hypothesis, findings showed that PEs toward students partially protect teachers from detrimental effect of negative ones on self-efficacy. As reported by Llorens et al. (2007), positive emotional states may help workers perceiving themselves more efficacious in performing daily tasks at work. Moreover, according to the broaden and build theory and its applications at work (Tugade and Fredrickson, 2004, 2007; Gloria et al., 2013), PEs may restore psychological resources (e.g., self-efficacy) despite the acknowledgment of NEs. How teachers’ PEs may have such a role? van der Want et al. (2018) stated that each teacher has a professional interpersonal standard, depending on personal beliefs and values, and related to the relationship with students. According to the authors, affective and cognitive appraisals about personal well-being and emotions and coping with stressful situations are represented accordingly to the interpersonal identity standards and guide teachers’ experience and expression of emotions. Accordingly, we have found that teachers recursively make use of the garden metaphor, reporting to be emotionally refueled by their students’ recognition (Buonomo and Fatigante, 2017). This point may clarify the bouncing back effect (Tugade and Fredrickson, 2004) found in this study: if teachers are willing to “accept” a contextual stressor, hoping to reach a more favorable outcome, then it is likely that PEs may help to build stronger efficacy beliefs regarding their teaching abilities.

Finally, contrarily to our second hypothesis, PEs toward professional role did not moderate the mediation effect shown in the study. This finding may be due to the characteristics of the Italian educational system itself. According to Bracci (2009), the web of accountability in Italian schools is getting confused because of several changes and reforms: in such a condition, it is likely that teachers take an individualistic point of view, giving more saliency and attention to the personal experience of teaching, than to the idea of being part of a specific group of professionals. Moreover, previous studies showed that being happy to be part of a school institution does not necessarily promote teachers’ efficacy beliefs (Malinen and Savolainen, 2016; Buonomo et al., 2017).

Regarding the limitations of the study, the use of longitudinal data is needed, to better address the mediation effect, giving more robust support to the findings, as mediations on cross-sectional data are known to be biased because of autoregressive effects (Maxwell and Cole, 2007). The impossibility to distinguish between “actual” predictors and results in cross-sectional designs (namely the impossibility to define a temporal lag between predictor and mediator, and mediator and outcome) weakens the inferences that can be made from our model. Moreover, the lack of a temporal lag didn’t allow to verify whether, as reported from previous studies (Canrinus and Fokkens-Bruinsma, 2011; Rodríguez-Sánchez et al., 2011; Lohbeck et al., 2018), feeling effective as a teacher may have influenced the level of PE and NE experienced in the classroom. For example, indeed, previous studies acknowledged that teachers with high self-efficacy are more prone to experience PE than NE with students, and vice versa (Tschannen-Moran et al., 1998). Furthermore, a distinction between the effects of trait- and state-emotions on self-efficacy should be done, as they differently impact teachers’ professional experiences (Goetz et al., 2015). Finally, a multimethod approach would give more information on how teachers perceive their emotions and how emotional experiences impact self-efficacy (Sutton and Wheatley, 2003).

Despite these limitations, our study shed new light on the effect of emotions on self-efficacy in teachers and have some practical implications concerning teachers’ training, regarding positive psychology interventions on school climate (Jennings and Greenberg, 2009). Improving teachers’ abilities to acknowledge positive exchanges and experiences within the school context may heighten their abilities to recognize personal attainments and successes as professionals. Moreover, our study suggests taking into account the organizational dimension of the teaching profession when intervening on teaching communities. Cultivating collaboration and sense of community within schools, indeed, may strengthening the effect of PEs: from one side, by increasing PEs during daily school life (Jennings and Greenberg, 2009), from the other, by improving positive perceptions of the teaching profession.

## Ethics Statement

The authors assert that all procedures contributing to this work comply with the ethical standards of the relevant national and institutional committees on human experimentation. All participants gave written informed consent in accordance with the Declaration of Helsinki. Ethical implications were evaluated by the Scientific Board of Libera Università Maria SS. Assunta (LUMSA), the body responsible for providing ethics approvals for studies, that approved the study.

## Author Contributions

IB designed and carried out the study. IB and CF contributed to the analysis of the results and to the writing of the manuscript. PB supervised the study design and the manuscript draft.

## Conflict of Interest Statement

The authors declare that the research was conducted in the absence of any commercial or financial relationships that could be construed as a potential conflict of interest.

## References

[B1] Aiken L. S. West S. G. (1991). *Multiple Regression: Testing and Interpreting Interactions.* Thousand Oaks, PA: SAGE.

[B2] Bandura A. (1986). The explanatory and predictive scope of self-efficacy theory. *J. Soc. Clin. Psychol.* 4 359–373. 10.1521/jscp.1986.4.3.359

[B3] Becker E. S. Keller M. M. Goetz T. Frenzel A. C. Taxer J. L. (2015). Antecedents of teachers’ emotions in the classroom: an intraindividual approach. *Front. Psychol.* 6:635. 10.3389/fpsyg.2015.00635 26042067 PMC4436560

[B4] Benevene P. Ittan M. M. Cortini M. (2018). Self-esteem and happiness as predictors of school teachers’ health: the mediating role of job satisfaction. *Front. Psychol.* 9:933. 10.3389/fpsyg.2018.00933 30065670 PMC6056906

[B5] Borrelli I. Benevene P. Fiorilli C. D’Amelio F. Pozzi G. (2014). Working conditions and mental health in teachers: a preliminary study. *Occup. Med.* 64 530–532. 10.1093/occmed/kqu108 25145485

[B6] Bracci E. (2009). Autonomy, responsibility and accountability in the Italian school system. *Crit. Perspect. Account.* 20 293–312. 10.1016/j.cpa.2008.09.001 24332234

[B7] Buonomo I. Fatigante M. (2017). *Effetto delle Relazioni nella Comunitá Scolastica sul Benessere Percepito degli Insegnanti [Effect of Relationship with Teaching Colleagues on Teacher Perceived Wellbeing]. Book of Abstracts of the 10th Italian Conference of Positive Psychology*. Available at: http://www.psicologiapositiva.it/wp-content/uploads/2017/06/book_of_abstracts_gnpp10.pdf (accessed July 2019).

[B8] Buonomo I. Fatigante M. Fiorilli C. (2017). The open psychology journal teachers’ burnout profile: risk and protective factors. *Open Psychol. J.* 10 190–201. 10.2174/1874350101710010190

[B9] Canrinus E. Fokkens-Bruinsma M. (2011). “Motivation to become a teacher and its relationships with teaching self-efficacy, professional commitment, and perceptions of the learning environment,” in *Paper Presented at the 24th International Congress for School Effectiveness and Improvement*, (Limassol).

[B10] Caprara G. V. Steca P. (2006). The contribution of self–regulatory efficacy beliefs in managing affect and family relationships to positive thinking and hedonic balance. *J. Soc. Clin. Psychol.* 25 603–627. 10.1521/jscp.2006.25.6.603

[B11] Chan D. W. (2004). Perceived emotional intelligence and self-efficacy among Chinese secondary school teachers in Hong Kong. *Pers. Individ. Differ.* 36 1781–1795. 10.1016/j.paid.2003.07.007

[B12] Diener E. Emmons R. A. (1984). The independence of positive and negative affect. *J. Pers. Soc. Psychol.* 47 1105–1117. 10.1037/0022-3514.47.5.1105 6520704

[B13] Diener E. Larsen R. J. Levine S. Emmons R. A. (1985). Intensity and frequency. Dimensions underlying positive and negative affect. *J. Pers. Soc. Psychol.* 48 1253–1265. 10.1037/0022-3514.48.5.1253 3998989

[B14] Diener E. Suh E. M. Lucas R. E. Smith H. L. (1999). Subjective well-being: three decades of progress. *Psychol. Bull.* 125 276–302. 10.1037/0033-2909.125.2.276 7630576

[B15] Fiorilli C. Pepe A. Buonomo I. Albanese O. (2017). At-risk teachers: the association between burnout levels and emotional appraisal processes. *Open Psychol. J.* 10 127–139. 10.2174/1874350101710010127

[B16] Fredrickson B. L. (2001). The role of positive emotions in positive psychology: the broaden-and-build theory of positive emotions. *Am. Psychol.* 56 218–226. 10.1037/0003-066X.56.3.218 11315248 PMC3122271

[B17] Fredrickson B. L. Losada M. F. (2005). Positive affect and the complex dynamics of human flourishing. *Am. Psychol.* 60 678–686. 10.1037/0003-066X.60.7.678 16221001 PMC3126111

[B18] Fredrickson B. L. Mancuso R. A. Branigan C. Tugade M. M. (2000). The undoing effect of positive emotions. *Motiv. Emot.* 24 237–258. 10.1023/A:1010796329158 21731120 PMC3128334

[B19] Frenzel A. C. Pekrun R. Goetz T. Daniels L. M. Durksen T. L. Becker-Kurz B. (2016). Measuring Teachers’ enjoyment, anger, and anxiety: the teacher emotions scales (TES). *Contemp. Educ. Psychol.* 46 148–163. 10.1016/j.cedpsych.2016.05.003

[B20] Gloria C. T. Faulk K. E. Steinhardt M. A. (2013). Positive affectivity predicts successful and unsuccessful adaptation to stress. *Motiv. Emot.* 37 185–193. 10.1007/s11031-012-9291-8

[B21] Goddard R. D. Hoy W. K. Hoy A. W. (2007). Collective efficacy beliefs: theoretical developments, empirical evidence, and future directions. *Educ. Res.* 33 3–13. 10.3102/0013189x033003003 19622800

[B22] Goetz T. Becker E. S. Bieg M. Keller M. M. Frenzel A. C. Hall N. C. (2015). The glass half empty: how emotional exhaustion affects the state-trait discrepancy in self-reports of teaching emotions. *PLoS One* 10:e0137441. 10.1371/journal.pone.0137441 26368911 PMC4569532

[B23] Gonzalez-Calvo G. Arias-Carballal M. (2017). A teacher’s personal-emotional identity and its reflection upon the development of his professional identity. *Qual. Rep.* 22 1693–1709.

[B24] González-Calvo G. Fernández-Balboa J. M. (2018). A qualitative analysis of the factors determining the quality of relations between a novice physical education teacher and his students’ families: implications for the development of professional identity. *Sport Educ. Soc.* 23 491–504. 10.1080/13573322.2016.1208164

[B25] Granziera H. Perera H. N. (2019). Relations among teachers’ self-efficacy beliefs, engagement, and work satisfaction: a social cognitive view. *Contemp. Educ. Psychol.* 58 75–84. 10.1016/j.cedpsych.2019.02.003

[B26] Hayes A. F. (2012). PROCESS: A Versatile Computational Tool for Observed Variable Mediation, Moderation, and Conditional Process Modeling. Available at: http://www.afhayes.com (accessed June 2019).

[B27] Holstein J. A. Gubrium J. F. (2000). *Constructing the Life Course*. Dix Hills, NY: General Hall.

[B28] Jennings P. A. Greenberg M. T. (2009). The prosocial classroom: teacher social and emotional competence in relation to student and classroom outcomes. *Rev. Educ. Res.* 79 491–525. 10.3102/0034654308325693

[B29] Kuppens P. Van Mechelen I. (2007). Interactional appraisal models for the anger appraisals of threatened self-esteem, other-blame, and frustration. *Cogn. Emot.* 21 56–77. 10.1080/02699930600562193

[B30] Llorens S. Schaufeli W. Bakker A. Salanova M. (2007). Does a positive gain spiral of resources, efficacy beliefs and engagement exist? *Comput. Human Behav.* 23 825–841. 10.1016/j.chb.2004.11.012

[B31] Lohbeck A. Hagenauer G. Frenzel A. C. (2018). Teachers’ self-concepts and emotions: conceptualization and relations. *Teach. Teach. Educ.* 70 111–120. 10.1016/j.tate.2017.11.001

[B32] Lyubomirsky S. King L. Diener E. (2005). The benefits of frequent positive affect: does happiness lead to success? *Psychol. Bull.* 131 803–855. 10.1037/0033-2909.131.6.803 16351326

[B33] Malinen O. P. Savolainen H. (2016). The effect of perceived school climate and teacher efficacy in behavior management on job satisfaction and burnout: a longitudinal study. *Teach. Teach. Educ.* 60 144–152. 10.1016/j.tate.2016.08.012

[B34] Maxwell S. E. Cole D. A. (2007). Bias in cross-sectional analyses of longitudinal mediation: partial and complete mediation under an autoregressive model. *Psychol. Methods* 12 23–44. 10.1080/00273171.2011.606716 17402810

[B35] McDonald R. P. (1999). *Test Theory: A Unified Treatment*. Mahwah, NJ: Lawrence Erlbaum Associates.

[B36] Mevarech Z. R. Maskit D. (2015). The teaching experience and the emotions it evokes. *Soc. Psychol. Educ.* 13 9286–9292. 10.1007/s11218-014-9286-2

[B37] Moè A. Pazzaglia F. Friso G. (2010). *MESI - Motivazione, Emozioni, Strategie e Insegnamento. Questionari Metacognitivi per gli insegnanti.* Trento: Erickson.

[B38] O’Connor K. E. (2008). “You choose to care”: teachers, emotions and professional identity. *Teach. Teach. Educ.* 24 117–126. 10.1016/j.tate.2006.11.008

[B39] OECD (2014). *TALIS 2013 Results: An International Perspective on Teaching and Learning.* Paris: OECD.

[B40] O’Malley A. L. Gregory J. B. (2011). Don’t be such a downer: using positive psychology to enhance the value of negative feedback. *Psychol. J.* 14 247–264. 10.1080/10887156.2011.621776

[B41] Palmer P. J. (1998). *The Courage to Teach: Exploring the Inner Landscape of a Teacher’s Life*. San Francisco: Jossey-Bass.

[B42] Preacher K. J. Rucker D. D. Hayes A. F. (2007). Addressing moderated mediation hypotheses: Theory, methods, and prescriptions. *Multivariate Behav. Res.* 42 185–227. 10.1080/00273170701341316 26821081

[B43] Revelle W. (2016). *R Package Psych: Procedures for Psychological, Psychometric, and Personality Research. R package version 1.7.8.*

[B44] Revelle W. Zinbarg R. E. (2009). Coefficients alpha, beta, omega, and the glb: comments on sijtsma. *Psychometrika* 74 145–154. 10.1007/s11336-008-9102-z 20037638

[B45] Roa-Tampe K. A. (2017). Teacher Evaluation from the standpoint of professional development: the chilean case. *Educ. Educ.* 20 41–61. 10.5294/edu.2017.20.1.3

[B46] Rodríguez-Sánchez A. Salanova M. Cifre E. Schaufeli W. B. (2011). When good is good: a virtuous circle of self-efficacy and flow at work among teachers. *Rev. Psicol. Soc.* 26 427–441. 10.1174/021347411797361257

[B47] Santisi G. Magnano P. Hichy Z. Ramaci T. (2014). Metacognitive strategies and work motivation in teachers: an empirical study. *Proc. Soc. Behav. Sci.* 116 1227–1231. 10.1016/j.sbspro.2014.01.373

[B48] Schutz P. A. Lee M. (2014). Teacher emotion, emotional labor and teacher identity. *Utr. Stud. Lang. Commun.* 27 169–186. 12343307

[B49] Staw B. M. Sutton R. I. Pelled L. H. (2008). Employee positive emotion and favorable outcomes at the workplace. *Organ. Sci.* 5 51–71. 10.1287/orsc.5.1.51

[B50] Sutton R. E. Harper E. (2009). “Teachers’ emotion regulation,” in *International Handbook of Research on Teachers and Teaching*, eds Saha L. J. Dworkin A. G. (Boston, MA: Springer), 389–401. 10.1007/978-0-387-73317-3_25

[B51] Sutton R. E. Wheatley K. F. (2003). Teachers’ emotions and teaching: a review of the literature and directions for future research. *Educ. Psychol. Rev.* 15 327–358. 10.1023/A:1026131715856 24499305

[B52] Thamm R. A. (2006). “The classification of emotions,” in *Handbook of the Sociology of Emotions*, eds Stets J. E. Turner J. H. (Boston, MA: Springer), 11–37. 10.1007/978-0-387-30715-2_2

[B53] Tracy J. L. Robins R. W. (2007). “The self in self-conscious emotions: a cognitive appraisal approach,” in *The Self-Conscious Emotions: Theory and Research*, eds Tracy J. L. Robins R. W. Tangney J. P. (New York, NY: Guilford Press), 3–20.

[B54] Tsai W. C. Chen C. C. Liu H. L. (2007). Test of a model linking employee positive moods and task performance. *J. Appl. Psychol.* 92 1570–1583. 10.1037/0021-9010.92.6.1570 18020797

[B55] Tschannen-Moran M. Hoy A. W. Hoy W. K. (1998). Teacher efficacy: its meaning and measure. *Rev. Educ. Res.* 68 202–248. 10.3102/00346543068002202 30462713

[B56] Tugade M. M. Fredrickson B. L. (2004). Resilient individuals use positive emotions to bounce back from negative emotional experiences. *J. Pers. Soc. Psychol.* 86 320–333. 10.1037/0022-3514.86.2.320 14769087 PMC3132556

[B57] Tugade M. M. Fredrickson B. L. (2007). Regulation of positive emotions: emotion regulation strategies that promote resilience. *J. Happiness Stud.* 8 311–333. 10.1007/s10902-006-9015-4

[B58] van der Want A. C. Schellings G. L. M. Mommers J. (2018). Experienced teachers dealing with issues in education: a career perspective. *Teach. Teach. Theory Pract.* 24 802–824. 10.1080/13540602.2018.1481024

[B59] Van Veen K. Sleegers P. (2006). How does it feel? Teachers’ emotions in a context of change. *J. Curric. Stud.* 38 85–111. 10.1080/00220270500109304

[B60] Watson D. (2000). Basic problems in positive mood regulation. *Psychol. Inq.* 11 205–209.

[B61] Wright T. A. Staw B. M. (1999). Affect and favorable work outcomes: two longitudinal tests of the happy-productive worker thesis. *J. Organ. Behav.* 20 1–23. 10.1002/(SICI)1099-1379(199901)20:1<1::AID-JOB885<3.0.CO;2-W

[B62] Xanthopoulou D. Bakker A. B. Demerouti E. Schaufeli W. B. (2012). A diary study on the happy worker: how job resources relate to positive emotions and personal resources. *Eur. J. Work Organ. Psychol.* 21 489–517. 10.1080/1359432X.2011.584386

[B63] Yin H.-B. Lee J. C. K. (2012). Be passionate, but be rational as well: emotional rules for Chinese teachers’ work. *Teach. Teach. Educ.* 28 56–65. 10.1016/j.tate.2011.08.005

[B64] Zee M. Koomen H. M. Y. (2016). Teacher self-efficacy and its effects on classroom processes, student academic adjustment, and teacher well-being. *Rev. Educ. Res.* 86 981–1015. 10.3102/0034654315626801

[B65] Zembylas M. (2003). Emotions and teacher identity: a poststructural perspective. *Teach. Teach. Theory Pract.* 9 213–238. 10.1080/13540600309378

